# Circadian clock disruption in neurodegenerative diseases: cause and effect?

**DOI:** 10.3389/fphar.2015.00029

**Published:** 2015-02-27

**Authors:** Erik S. Musiek

**Affiliations:** Hope Center for Neurological Disorders and Charles F. and Joanne Knight Alzheimer’s Disease Research Center, Department of Neurology, Washington University School of Medicine in St. Louis, Saint Louis, MO, USA

**Keywords:** circadian clock, Bmal1, Per2, neurodegeneration, Alzheimer disease, Huntington disease

## Abstract

Disturbance of the circadian system, manifested as disrupted daily rhythms of physiologic parameters such as sleep, activity, and hormone secretion, has long been observed as a symptom of several neurodegenerative diseases, including Alzheimer disease. Circadian abnormalities have generally been considered consequences of the neurodegeneration. Recent evidence suggests, however, that circadian disruption might actually contribute to the neurodegenerative process, and thus might be a modifiable cause of neural injury. Herein we will review the evidence implicating circadian rhythms disturbances and clock gene dysfunction in neurodegeneration, with an emphasis on future research directions and potential therapeutic implications for neurodegenerative diseases.

## INTRODUCTION

Numerous studies over the past 30 years have described a wide variety of circadian and sleep-wake cycle aberrations which occur in aging and neurodegenerative diseases (Ju et al., 2014; Videnovic et al., 2014a). Many behavioral and physiologic processes oscillate with a 24 h period, including sleep–wake, activity, body temperature, blood pressure, and hormone secretion. These circadian rhythms are frequently disrupted in patients with neurodegenerative disease, including Alzheimer disease (AD), Parkinson disease (PD), Huntington disease (HD). Systemic circadian rhythms in mice and humans are maintained via the function of the body’s master clock in the suprachiasmatic nucleus (SCN), which receives input from the retina and synchronizes oscillations in peripheral organs to the light:dark cycle. On a cellular level, circadian rhythms are generated by a transcriptional–translational feedback loop consisting of the bHLH/PAS transcription factors BMAL1 and CLOCK, which heterodimerize and drive transcription of many genes, including their own negative feedback repressors, including PERIOD (Per) and CRYPTOCHROME (Cry) and REVERB genes, which repress BMAL1/CLOCK-mediated transcription (Mohawk et al., 2012). This transcriptional machinery, which we will refer to herein as the core circadian clock, is present in most cells in the body, including neurons and astrocytes in the SCN and throughout the brain. The core circadian clock regulates the circadian expression of thousands of genes in a tissue-specific manner, and is a major regulator of cellular metabolism, stress response, and many other functions (Bass and Takahashi, 2010; Evans and Davidson, 2013). While whole-organism rhythms are known to be disrupted in many neurodegenerative diseases, far less information exists regarding specific alterations in clock protein expression and function in these conditions. Furthermore, it remains unclear if or how circadian disruption might influence the neurodegenerative process itself, or if the core clock represents a reasonable therapeutic target for the treatment or prevention of neurodegeneration. We will focus on these issues in this review.

## PART 1: IS THERE EVIDENCE OF CORE CIRCADIAN CLOCK DYSFUNCTION IN NEURODEGENERATIVE DISEASES?

In AD, both sleep and circadian dysfunction are commonly reported. While sleep disturbances in AD are beyond the scope of this discussion and have been reviewed elsewhere (Ju et al., 2014; Peter-Derex et al., 2014), it is important to mention that subtle sleep disturbances appear to occur early in the disease process and may predict amyloid-beta (Aβ) plaque pathology and precede subsequent development of clinical dementia (Ju et al., 2013; Lim et al., 2013a; Spira et al., 2013). Disrupted circadian rhythms in activity, physiologic parameters, and melatonin secretion have been reported in AD reported by several groups (Witting et al., 1990; Skene and Swaab, 2003; Hatfield et al., 2004; Wu et al., 2006; Hu et al., 2009; Coogan et al., 2013). A proposed mechanism of circadian dysfunction in AD is degeneration of the SCN, as loss of critical vasopressin and vasoactive intestinal peptide (VIP)-expressing neurons in this region has been reported in AD patients (Swaab et al., 1985; Zhou et al., 1995; Farajnia et al., 2012). Transcriptional analysis of postmortem human brain tissue has revealed detectable oscillations in core clock genes in various brain regions based on time of death, and shown that in AD brains the phase of oscillation is dysregulated between various regions (Cermakian et al., 2011; Lim et al., 2013b). Rhythms in whole-genome DNA methylation could also be detected which appear to become less robust with age or in AD brains (Lim et al., 2014). Circadian oscillation of clock genes in the human pineal gland was disrupted even at very early pathological stages of AD, mirroring loss of rhythmic melatonin secretion in AD patients (Skene and Swaab, 2003; Wu et al., 2006). Thus, in human AD there is evidence of disturbed rhythms of clock gene expression which appear to begin early in the disease course.

Animal models of AD also exhibit disturbances of behavioral and physiologic circadian rhythms. In mice, these disturbances appear to correlate with the degree of amyloid plaque burden, and can in some cases be rescued with anti-Aβ immunotherapy, suggesting that aggregated forms of Aβ might disrupt clock mechanisms (Wisor et al., 2005; Sterniczuk et al., 2010; Duncan et al., 2012; Roh et al., 2012). However, while one study described damped expression of *Per2* in the SCN of APP-PS1 transgenic mice (Duncan et al., 2012), more detailed molecular analysis of clock gene function in AD mouse models is lacking. In *Drosophila*, two groups have found that pan-neuronal expression of arctic mutant human Aβ causes marked degradation of behavioral circadian rhythms, despite preserved clock gene oscillation in the central pacemaker cells (Chen et al., 2014; Long et al., 2014). Chen et al. (2014) found that restricted Aβ expression in central clock (PDF) neurons did not disrupt clock gene oscillation or cause behavioral arrhythmicity, which Aβ expression in glia surrounding the clock neurons did both. Thus, in flies, Aβ does not directly disrupt clock gene function in the central pacemaker, but acts more peripherally (and perhaps on glia) to disrupt behavioral rhythms. While the biological relevance of this fly models of Aβ toxicity to humans is debatable, these findings provide leads for further research in mammalian models.

In the case of PD, the second most common age-related neurodegenerative condition, there is ample evidence of disrupted circadian rhythms and sleep–wake disturbance in humans and mouse models (Videnovic and Golombek, 2013). PD patients exhibit progressive disruption of activity rhythms (Niwa et al., 2011), as well as damped circadian oscillation of both melatonin release and *Bmal1* expression in peripheral blood monocytes (Cai et al., 2010; Breen et al., 2014; Videnovic et al., 2014b). Fly and mouse models of PD which expresses mutant human α-synuclein, a protein implicated in PD pathogenesis, develop behavioral circadian disruption early in its disease course (Gajula Balija et al., 2011; Kudo et al., 2011a). Synuclein transgenic mice display normal *Per2* oscillation in the SCN, but have damped electrical output from the SCN, again suggesting disordered SCN function or synchrony (Kudo et al., 2011a).

Huntington disease, unlike AD and PD, is an autosomal disorder caused by trinucleotide expansion within the *huntingtin* gene. Neurodegeneration occurs earlier in HD patients and initially involves the basal ganglia. Sleep and circadian rhythm dysfunction are common in HD (Morton et al., 2005; Aziz et al., 2010), though there is a paucity of studies on clock gene expression and function in human HD. Several mouse models of HD, which express expanded human *huntingtin*, develop pronounced impairment in behavioral circadian rhythms (Morton et al., 2005; Kudo et al., 2011b). The R6/2 line exhibits behavioral arrhythmicity as well as dysregulated clock gene oscillation *in vivo* in the liver and SCN (Morton et al., 2005; Maywood et al., 2010). Interestingly, clock gene oscillation appears to be normal in liver or SCN explants from R6/2 mice, suggesting that some other aspect of the internal milieu of the R6/2 mouse is causing arrhythmicity *in vivo* (Pallier et al., 2007; Maywood et al., 2010). In another HD mouse line (BACHD) the rhythmicity of electrical output of the SCN was disrupted, while the oscillation of *Per2* transcription was grossly intact (Kudo et al., 2011b). These findings suggest support the idea that dysfunction of the neural networks within the SCN, rather than the cell-intrinsic clock gene oscillation, underlies circadian impairment in HD model mice. Accordingly, decreased expression of the neuropeptide VIP and the VIP receptor VPAC2, which are critical for SCN function (Aton et al., 2005), was also observed in R6/2 mice (Fahrenkrug et al., 2007). It appears that in HD mice, disrupted peripheral rhythms may adversely impact SCN function, leading to further systemic circadian arrhythmicity, though the details of this mechanism are still being explored.

## PART 2: IS THERE EVIDENCE THAT CLOCK DISRUPTION EXACERBATES NEURODEGENERATION?

While circadian disturbances in aging and neurodegenerative diseases have been duly noted, a key question is whether these disturbances influence disease pathology. This question is much more difficult to examine, and has received significantly less attention. In *Drosophila*, levels of oxidative stress markers, as well as cellular content of the critical antioxidant glutathione show circadian oscillation which is dependent on the clock gene Period (*Per*, Krishnan et al., 2008; Beaver et al., 2012). *Per* deletion exacerbates oxidative injury and shortens lifespan in *Drosophila* (Krishnan et al., 2008, 2009). Disruption of clock function via *Per* deletion also accelerates neurodegeneration in flies bearing a carbonyl reductase mutation which causes oxidative injury to neurons (Krishnan et al., 2012). However, in fly models of Aβ pathology which express different human Aβ isoforms, *Per* deletion did not impact neurodegeneration or behavior, though lifespan was decreased. Conversely, levels of Cryptochrome (*Cry*), which serves as a light-responsive modulator of clock function in *Drosophila*, decline in parallel with damped circadian rhythms in old flies, while *Cry* overexpression restores robust rhythms and enhances lifespan (Rakshit and Giebultowicz, 2013). Thus, the clock genes *Per* and *Cry* clearly appear to contribute to regulation of brain aging and neurodegeneration in fly models.

In mice, evidence linking circadian dysfunction and neurodegeneration is emerging. Chronic disruption of circadian rhythms in mice via housing in 20:4 light:dark conditions leads to decreased neuronal dendritic arborization and cognitive deficits, demonstrating that disturbed circadian rhythms have negative implications for the brain, though the exact degree of clock gene dysregulation and the role of other factors such as stress are unknown (Karatsoreos et al., 2011). Accordingly, studies in rats and hamsters have demonstrated cognitive impairment and decreased hippocampal neurogenesis following chronic “jet lag” protocols, during which circadian rhythms are disrupted by frequent shifting of the light:dark cycle (Gibson et al., 2010; Kott et al., 2012). Mechanistically, RevErbα-mediated regulation of fatty acid binding protein 7 (*Fabp7*), both of which are strongly controlled by the core clock, has been implicated in neurogenesis (Schnell et al., 2014). Similarly, RevErbα shows dynamic activity-dependent regulation in the dendrites of hippocampal neurons and interacts with oligophrenin-1, a regulator of dendritic spines (Valnegri et al., 2011). In a mouse model of AD, Aβ levels in the brain interstitial fluid show pronounced circadian oscillation (Kang et al., 2009), though it unclear if this is a direct effect of the sleep–wake cycle or may be more directly clock-mediated.

In order to gain some appreciation of the role of clock genes in maintaining brain homeostasis, our group performed functional and neuropathologic analysis of mice with global deletion of *Bmal1*. *Bmal1* knockout (KO) mice developed marked astrogliosis which was evident by 2 months of age and progressed to involve the entirety of the cortex, striatum, and hippocampus (Musiek et al., 2013). These mice also had increased levels of oxidative damage in the cortex, and exhibited spontaneous degeneration of presynaptic terminals and diminished cortical functional connectivity. We found a similar phenotype in *Clock;Npas2* double KO mice, which like *Bmal1* KOs have completely disabled core clock transcriptional function (DeBruyne et al., 2007), but not in *Per1/Per2* double mutant mice, which are arrhythmic but have intact core clock-mediated transcription (Bae et al., 2001). We subsequently generated brain-specific *Bmal1* KO mice which have preserved SCN *Bmal1* expression and intact systemic circadian activity and sleep rhythms, but disrupted BMAL1-mediated transcription in cortical, striatal, and hippocampal neurons and astrocytes. These mice also developed severe astrogliosis, suggesting that positive-limb core clock function is required locally in neurons and/or astrocytes to prevent pathology, independent of the SCN or sleep–wake cycle (Musiek et al., 2013). Finally, we found that neurodegeneration induced by the mitochondrial complex II inhibitor 3-nitropropionic acid, which has been used to model HD (Beal et al., 1995), was exacerbated in *Bmal1* hemizygous mice, which have intact systemic rhythms but only half the normal level of BMAL1 protein expression in the brain. Thus, it appears that the core clock transcriptional machinery plays a critical role in protecting the brain from oxidative injury, and that this function is not entirely dictated by systemic circadian rhythms. Accordingly, we found that *Bmal1* directly regulates the transcription of several important redox defense genes in the brain, including *Nqo1* and *Aldh2*. We are currently working to identify novel mechanisms by which the core clock mediates neuroprotection or neurodegeneration, and to understand the relative importance of systemic circadian rhythms and clock gene oscillation versus static transcriptional function in this process.

Conversely, the effect of improving circadian function on pathology in a mouse model of neurologic disease has been demonstrated, at least initially. In the aforementioned R6/2 mouse model of HD, pharmacologic induction of rhythmic sleep normalized *Per2* oscillation in the SCN and lead to improvements in cognition (Pallier et al., 2007; Pallier and Morton, 2009). Furthermore, when food was provided to these mice only during a strategic window in the circadian cycle, rhythms in behavior were restored and metabolic abnormalities in these mice improved, suggesting that synchronizing the food-entrainable oscillator could overcome the circadian defect. Thus, correcting peripheral rhythms by imposing circadian sleep or feeding schedules can mitigate cognitive impairment in a mouse model of HD (Maywood et al., 2010). The application of these methods, or more specific molecular targeting of the core clock or its outputs, now needs to be evaluated in other animal models of neurodegeneration.

Human data demonstrating a contributory effect of circadian disruption to neurodegeneration is scarce, though several encouraging findings have emerged. Two observational studies examining young female flight attendants who routinely flew across multiple time zones found that the group that those afforded shorter recovery time between cross-time zone flights (who thus experienced more severe circadian disruption) had higher cortisol levels, smaller temporal lobe volumes on MRI, and performed more poorly on hippocampal-based cognitive testing than ground crew members or other flight attendants with less severe jet lag exposure (Cho et al., 2000; Cho, 2001). In the field of AD, a small amount of human data now also exists which supports a role for circadian disruption in disease pathogenesis. Three separate genetic polymorphism in the *Clock* gene have been linked to increased risk of AD in Han Chinese populations (Chen et al., 2013a,b; Yang et al., 2013), though these findings have not been reported by other large AD genetics consortiums. An epidemiologic study of daily activity data from of over 1,200 initially cognitively-normal older women demonstrated that diminished circadian rhythm amplitude, robustness, or phase delay were associated with increased risk of developing dementia during the 5 year follow-up period (Tranah et al., 2011). On a more mechanistic level, circadian oscillations in the level of the Aβ in cerebrospinal fluid of older adults has been described and suggest possible regulation of Aβ metabolism by the circadian clock, though it does not demonstrate a clear role for these oscillations in the disease process (Kang et al., 2009; Huang et al., 2012). Further research into circadian function in prodromal AD and other neurodegenerative disease, and how this relates to disease risk or progression is needed.

## CONCLUSIONS AND THERAPEUTIC PERSPECTIVES

Taken in total, there is promising early data but not iron-clad proof that circadian clock disruption contributes to the pathogenesis of age-related neurodegenerative diseases. Two major challenges in this area are apparent. First, distinguishing the specific effects of alterations in sleep from those of the circadian clock is difficult but necessary. Activity data (actigraphy) in humans is often used as a biomarker of sleep or circadian rhythms, and the two processes are often lumped together, obscuring specific conclusions about either. Of further concern is the fact that disrupting sleep impacts core clock protein function (Mongrain et al., 2011), while deleting clock genes also alters sleep (Laposky et al., 2005). Disentangling these two processes is important if we hope to identify specific downstream pharmacologic targets from either pathway to treat or prevent neurodegenerative diseases.

The second major challenge involves distinguishing the specific importance of circadian oscillation versus the “static” function of clock proteins in the brain. While circadian oscillations are observed in thousands of transcripts in many tissues, including the brain, the physiological relevance of these oscillations remains in many cases unclear (Zhang et al., 2014). Clock proteins exert various functions in cells, some of which may have less dependence on these oscillations. Ultimately, the function of clock proteins is never completely disengaged from their oscillation, as the BMAL1/CLOCK DNA binding shows clear circadian variation (Koike et al., 2012), but the relative importance of rhythmic versus static function remains a key therapeutic question. If restoration of robust systemic oscillations is the therapeutic goal, then therapies might target the SCN. Vasopressin V1a and V1b, as well as VIP VPAC2 receptors, play critical roles in synchronizing SCN neurons and their response to phase shift, and thus might represent tractable therapeutic targets to optimize systemic rhythms (Aton et al., 2005; An et al., 2013; Kudo et al., 2013; Yamaguchi et al., 2013). Behavioral manipulations such as imposed light:dark exposure, timed melatonin treatment, or rhythmic meal schedules might have shown promise in HD mouse models and might also be considered (Pallier et al., 2007; Maywood et al., 2010). Finally, novel small molecule modulators of clock oscillation are being developed which alter the period, amplitude, or frequency of SCN output (Hirota et al., 2010; Chen et al., 2012). However, if bolstering clock gene expression outside the SCN is the more advantageous strategy, then a new set of therapies would need to be developed. In this case, downstream neuroprotective targets of the core clock would need to be identified and screening pursued to identify compounds or strategies which enhance core clock transcription of these protective downstream targets. Because the clock serves as an orchestrator of a multitude of biological processes, there is great potential for clock-targeted therapeutics to simultaneously ameliorate multiple pathologic aspects of complex neurodegenerative diseases. Thus, it is important to more fully understand the mechanisms by which the circadian clock regulates brain function and neurodegeneration, such that rational strategies to target the clock for neuroprotection can be devised.

### Conflict of Interest Statement

The author declares that the research was conducted in the absence of any commercial or financial relationships that could be construed as a potential conflict of interest.

## References

[B1] An S. Harang R. Meeker K. Granados-Fuentes D. Tsai C. A. Mazuski C. (2013). A neuropeptide speeds circadian entrainment by reducing intercellular synchrony. Proc. Natl. Acad. Sci. U.S.A. 110, E4355–E4361. 10.1073/pnas.1307088110 24167276 PMC3832006

[B2] Aton S. J. Colwell C. S. Harmar A. J. Waschek J. Herzog E. D. (2005). Vasoactive intestinal polypeptide mediates circadian rhythmicity and synchrony in mammalian clock neurons. Nat. Neurosci. 8, 476–483. 10.1038/nn1419 15750589 PMC1628303

[B3] Aziz N. A. Anguelova G. V. Marinus J. Lammers G. J. Roos R. A. (2010). Sleep and circadian rhythm alterations correlate with depression and cognitive impairment in Huntington’s disease. Parkinsonism Relat. Disord. 16, 345–350. 10.1016/j.parkreldis.2010.02.009 20236854

[B4] Bae K. Jin X. Maywood E. S. Hastings M. H. Reppert S. M. Weaver D. R. (2001). Differential functions of *mPer1*, *mPer2*, and *mPer3* in the SCN circadian clock. Neuron 30, 525–536 10.1016/S0896-6273(01)00302-6 11395012

[B5] Bass J. Takahashi J. S. (2010). Circadian integration of metabolism and energetics. Science 330, 1349–1354. 10.1126/science.1195027 21127246 PMC3756146

[B6] Beal M. F. Ferrante R. J. Henshaw R. Matthews R. T. Chan P. H. Kowall N. W. (1995). 3-Nitropropionic acid neurotoxicity is attenuated in copper/zinc superoxide dismutase transgenic mice. J. Neurochem. 65, 919–922. 10.1046/j.1471-4159.1995.65020919.x 7616254

[B7] Beaver L. M. Klichko V. I. Chow E. S. Kotwica-Rolinska J. Williamson M. Orr W. C. (2012). Circadian regulation of glutathione levels and biosynthesis in *Drosophila melanogaster*. PLoS ONE 7:e50454. 10.1371/journal.pone.0050454 23226288 PMC3511579

[B8] Breen D. P. Vuono R. Nawarathna U. Fisher K. Shneerson J. M. Reddy A. B. (2014). Sleep and circadian rhythm regulation in early Parkinson disease. JAMA Neurol. 71, 589–595. 10.1001/jamaneurol.2014.65 24687146 PMC4119609

[B9] Cai Y. Liu S. Sothern R. B. Xu S. Chan P. (2010). Expression of clock genes *Per1* and *Bmal1* in total leukocytes in health and Parkinson’s disease. Eur. J. Neurol. 17, 550–554. 10.1111/j.1468-1331.2009.02848.x 19912323

[B10] Cermakian N. Lamont E. W. Boudreau P. Boivin D. B. (2011). Circadian clock gene expression in brain regions of Alzheimer’s disease patients and control subjects. J. Biol. Rhythms 26, 160–170. 10.1177/0748730410395732 21454296

[B11] Chen H. F. Huang C. Q. You C. Wang Z. R. Si-Qing H. (2013a). Polymorphism of CLOCK gene rs 4580704 C > G is associated with susceptibility of Alzheimer’s disease in a Chinese population. Arch. Med. Res. 44, 203–207. 10.1016/j.arcmed.2013.01.002 23357097

[B12] Chen Q. Huang C. Q. Hu X. Y. Li S. B. Zhang X. M. (2013b). Functional CLOCK gene rs1554483 G/C polymorphism is associated with susceptibility to Alzheimer’s disease in the Chinese population. J. Int. Med. Res. 41, 340–346. 10.1177/0300060513476430 23781009

[B13] Chen K. F. Possidente B. Lomas D. A. Crowther D. C. (2014). The central molecular clock is robust in the face of behavioural arrhythmia in a *Drosophila* model of Alzheimer’s disease. Dis. Model. Mech. 7, 445–458. 10.1242/dmm.014134 24574361 PMC3974455

[B14] Chen Z. Yoo S. H. Park Y. S. Kim K. H. Wei S. Buhr E. (2012). Identification of diverse modulators of central and peripheral circadian clocks by high-throughput chemical screening. Proc. Natl. Acad. Sci. U.S.A. 109, 101–106. 10.1073/pnas.1118034108 22184224 PMC3252927

[B15] Cho K. (2001). Chronic ‘jet lag’ produces temporal lobe atrophy and spatial cognitive deficits. Nat. Neurosci. 4, 567–568. 10.1038/88384 11369936

[B16] Cho K. Ennaceur A. Cole J. C. Suh C. K. (2000). Chronic jet lag produces cognitive deficits. J. Neurosci. 20, 1–5. 10704520 10.1523/JNEUROSCI.20-06-j0005.2000 PMC6772481

[B17] Coogan A. N. Schutova B. Husung S. Furczyk K. Baune B. T. Kropp P. (2013). The circadian system in Alzheimer’s disease: disturbances, mechanisms, and opportunities. Biol. Psychiatry 74, 333–339. 10.1016/j.biopsych.2012.11.021 23273723

[B18] DeBruyne J. P. Weaver D. R. Reppert S. M. (2007). CLOCK and NPAS2 have overlapping roles in the suprachiasmatic circadian clock. Nat. Neurosci. 10, 543–545. 10.1038/nn1884 17417633 PMC2782643

[B19] Duncan M. J. Smith J. T. Franklin K. M. Beckett T. L. Murphy M. P. St Clair D. K. (2012). Effects of aging and genotype on circadian rhythms, sleep, and clock gene expression in APPxPS1 knock-in mice, a model for Alzheimer’s disease. Exp. Neurol. 236, 249–258. 10.1016/j.expneurol.2012.05.011 22634208

[B20] Evans J. A. Davidson A. J. (2013). Health consequences of circadian disruption in humans and animal models. Prog. Mol. Biol. Transl. Sci. 119, 283–323. 10.1016/B978-0-12-396971-2.00010-5 23899601

[B21] Fahrenkrug J. Popovic N. Georg B. Brundin P. Hannibal J. (2007). Decreased VIP and VPAC2 receptor expression in the biological clock of the R6/2 Huntington’s disease mouse. J. Mol. Neurosci. 31, 139–148. 10.1385/JMN/31:02:139 17478887

[B22] Farajnia S. Michel S. Deboer T. Vanderleest H. T. Houben T. Rohling J. H. (2012). Evidence for neuronal desynchrony in the aged suprachiasmatic nucleus clock. J. Neurosci. 32, 5891–5899. 10.1523/JNEUROSCI.0469-12.2012 22539850 PMC6703600

[B23] Gajula Balija M. B. Griesinger C. Herzig A. Zweckstetter M. Jackle H. (2011). Pre-fibrillar *α*-synuclein mutants cause Parkinson’s disease-like non-motor symptoms in *Drosophila*. PLoS ONE 6:e24701. 10.1371/journal.pone.0024701 21931820 PMC3169624

[B24] Gibson E. M. Wang C. Tjho S. Khattar N. Kriegsfeld L. J. (2010). Experimental ‘jet lag’ inhibits adult neurogenesis and produces long-term cognitive deficits in female hamsters. PLoS ONE 5:e15267. 10.1371/journal.pone.0015267 21152025 PMC2995744

[B25] Hatfield C. F. Herbert J. Van Someren E. J. Hodges J. R. Hastings M. H. (2004). Disrupted daily activity/rest cycles in relation to daily cortisol rhythms of home-dwelling patients with early Alzheimer’s dementia. Brain 127, 1061–1074. 10.1093/brain/awh129 14998915

[B26] Hirota T. Lee J. W. Lewis W. G. Zhang E. E. Breton G. Liu X. (2010). High-throughput chemical screen identifies a novel potent modulator of cellular circadian rhythms and reveals CKI*α* as a clock regulatory kinase. PLoS Biol. 8:e1000559. 10.1371/journal.pbio.1000559 21179498 PMC3001897

[B27] Hu K. Van Someren E. J. Shea S. A. Scheer F. A. (2009). Reduction of scale invariance of activity fluctuations with aging and Alzheimer’s disease: involvement of the circadian pacemaker. Proc. Natl. Acad. Sci. U.S.A. 106, 2490–2494. 10.1073/pnas.0806087106 19202078 PMC2650290

[B28] Huang Y. Potter R. Sigurdson W. Santacruz A. Shih S. Ju Y. E. (2012). Effects of age and amyloid deposition on A*β* dynamics in the human central nervous system. Arch. Neurol. 69, 51–58. 10.1001/archneurol.2011.235 21911660 PMC3254706

[B29] Ju Y. E. Lucey B. P. Holtzman D. M. (2014). Sleep and Alzheimer disease pathology—a bidirectional relationship. Nat. Rev. Neurol. 10, 115–119. 10.1038/nrneurol.2013.269 24366271 PMC3979317

[B30] Ju Y. E. Mcleland J. S. Toedebusch C. D. Xiong C. Fagan A. M. Duntley S. P. (2013). Sleep quality and preclinical Alzheimer disease. JAMA Neurol. 70, 587–593. 10.1001/jamaneurol.2013.2334 23479184 PMC3676720

[B31] Kang J. E. Lim M. M. Bateman R. J. Lee J. J. Smyth L. P. Cirrito J. R. (2009). Amyloid-*β* dynamics are regulated by orexin and the sleep–wake cycle. Science 326, 1005–1007. 10.1126/science.1180962 19779148 PMC2789838

[B32] Karatsoreos I. N. Bhagat S. Bloss E. B. Morrison J. H. Mcewen B. S. (2011). Disruption of circadian clocks has ramifications for metabolism, brain, and behavior. Proc. Natl. Acad. Sci. U.S.A. 108, 1657–1662. 10.1073/pnas.1018375108 21220317 PMC3029753

[B33] Koike N. Yoo S. H. Huang H. C. Kumar V. Lee C. Kim T. K. (2012). Transcriptional architecture and chromatin landscape of the core circadian clock in mammals. Science 338, 349–354. 10.1126/science.1226339 22936566 PMC3694775

[B34] Kott J. Leach G. Yan L. (2012). Direction-dependent effects of chronic “jet-lag” on hippocampal neurogenesis. Neurosci. Lett. 515, 177–180. 10.1016/j.neulet.2012.03.048 22465247

[B35] Krishnan N. Davis A. J. Giebultowicz J. M. (2008). Circadian regulation of response to oxidative stress in *Drosophila melanogaster*. Biochem. Biophys. Res. Commun. 374, 299–303. 10.1016/j.bbrc.2008.07.011 18627767 PMC2553425

[B36] Krishnan N. Kretzschmar D. Rakshit K. Chow E. Giebultowicz J. M. (2009). The circadian clock gene period extends healthspan in aging *Drosophila melanogaster*. Aging (Albany NY) 1, 937–948. 20157575 10.18632/aging.100103 PMC2815745

[B37] Krishnan N. Rakshit K. Chow E. S. Wentzell J. S. Kretzschmar D. Giebultowicz J. M. (2012). Loss of circadian clock accelerates aging in neurodegeneration-prone mutants. Neurobiol. Dis. 45, 1129–1135. 10.1016/j.nbd.2011.12.034 22227001 PMC3291167

[B38] Kudo T. Loh D. H. Truong D. Wu Y. Colwell C. S. (2011a). Circadian dysfunction in a mouse model of Parkinson’s disease. Exp. Neurol. 232, 66–75. 10.1016/j.expneurol.2011.08.003 21864527

[B39] Kudo T. Schroeder A. Loh D. H. Kuljis D. Jordan M. C. Roos K. P. (2011b). Dysfunctions in circadian behavior and physiology in mouse models of Huntington’s disease. Exp. Neurol. 228, 80–90. 10.1016/j.expneurol.2010.12.011 21184755 PMC4346330

[B40] Kudo T. Tahara Y. Gamble K. L. Mcmahon D. G. Block G. D. Colwell C. S. (2013). Vasoactive intestinal peptide produces long-lasting changes in neural activity in the suprachiasmatic nucleus. J. Neurophysiol. 110, 1097–1106. 10.1152/jn.00114.2013 23741043 PMC4073931

[B41] Laposky A. Easton A. Dugovic C. Walisser J. Bradfield C. Turek F. (2005). Deletion of the mammalian circadian clock gene BMAL1/Mop3 alters baseline sleep architecture and the response to sleep deprivation. Sleep 28, 395–409. 16171284 10.1093/sleep/28.4.395

[B42] Lim A. S. Kowgier M. Yu L. Buchman A. S. Bennett D. A. (2013a). Sleep fragmentation and the risk of incident Alzheimer’s disease and cognitive decline in older persons. Sleep 36, 1027–1032. 10.5665/sleep.2802 23814339 PMC3669060

[B43] Lim A. S. Myers A. J. Yu L. Buchman A. S. Duffy J. F. De Jager P. L. (2013b). Sex difference in daily rhythms of clock gene expression in the aged human cerebral cortex. J. Biol. Rhythms 28, 117–129. 10.1177/0748730413478552 23606611 PMC3774838

[B44] Lim A. S. Srivastava G. P. Yu L. Chibnik L. B. Xu J. Buchman A. S. (2014). 24-hour rhythms of DNA methylation and their relation with rhythms of RNA expression in the human dorsolateral prefrontal cortex. PLoS Genet. 10:e1004792. 10.1371/journal.pgen.1004792 25375876 PMC4222754

[B45] Long D. M. Blake M. R. Dutta S. Holbrook S. D. Kotwica-Rolinska J. Kretzschmar D. (2014). Relationships between the circadian system and Alzheimer’s disease-like symptoms in *Drosophila*. PLoS ONE 9:e106068. 10.1371/journal.pone.0106068 25171136 PMC4149435

[B46] Maywood E. S. Fraenkel E. Mcallister C. J. Wood N. Reddy A. B. Hastings M. H. (2010). Disruption of peripheral circadian timekeeping in a mouse model of Huntington’s disease and its restoration by temporally scheduled feeding. J. Neurosci. 30, 10199–10204. 10.1523/JNEUROSCI.1694-10.2010 20668203 PMC6633357

[B47] Mohawk J. A. Green C. B. Takahashi J. S. (2012). Central and peripheral circadian clocks in mammals. Annu. Rev. Neurosci. 35, 445–462. 10.1146/annurev-neuro-060909-153128 22483041 PMC3710582

[B48] Mongrain V. La Spada F. Curie T. Franken P. (2011). Sleep loss reduces the DNA-binding of BMAL1, CLOCK, and NPAS2 to specific clock genes in the mouse cerebral cortex. PLoS ONE 6:e26622. 10.1371/journal.pone.0026622 22039518 PMC3200344

[B49] Morton A. J. Wood N. I. Hastings M. H. Hurelbrink C. Barker R. A. Maywood E. S. (2005). Disintegration of the sleep–wake cycle and circadian timing in Huntington’s disease. J. Neurosci. 25, 157–163. 10.1523/JNEUROSCI.3842-04.2005 15634777 PMC6725210

[B50] Musiek E. S. Lim M. M. Yang G. Bauer A. Q. Qi L. Lee Y. (2013). Circadian clock proteins regulate neuronal redox homeostasis and neurodegeneration. J. Clin. Invest. 123, 5389–5400. 10.1172/JCI70317 24270424 PMC3859381

[B51] Niwa F. Kuriyama N. Nakagawa M. Imanishi J. (2011). Circadian rhythm of rest activity and autonomic nervous system activity at different stages in Parkinson’s disease. Auton. Neurosci. 165, 195–200. 10.1016/j.autneu.2011.07.010 21871844

[B52] Pallier P. N. Maywood E. S. Zheng Z. Chesham J. E. Inyushkin A. N. Dyball R. (2007). Pharmacological imposition of sleep slows cognitive decline and reverses dysregulation of circadian gene expression in a transgenic mouse model of Huntington’s disease. J. Neurosci. 27, 7869–7878. 10.1523/JNEUROSCI.0649-07.2007 17634381 PMC6672877

[B53] Pallier P. N. Morton A. J. (2009). Management of sleep/wake cycles improves cognitive function in a transgenic mouse model of Huntington’s disease. Brain Res. 1279, 90–98. 10.1016/j.brainres.2009.03.072 19450569

[B54] Peter-Derex L. Yammine P. Bastuji H. Croisile B. (2014). Sleep and Alzheimer’s disease. Sleep Med. Rev. 19C, 29–38 10.1016/j.smrv.2014.03.007 24846773

[B55] Rakshit K. Giebultowicz J. M. (2013). Cryptochrome restores dampened circadian rhythms and promotes healthspan in aging *Drosophila*. Aging Cell 12, 752–762. 10.1111/acel.12100 23692507 PMC3772985

[B56] Roh J. H. Huang Y. Bero A. W. Kasten T. Stewart F. R. Bateman R. J. (2012). Disruption of the sleep–wake cycle and diurnal fluctuation of *β*-amyloid in mice with Alzheimer’s disease pathology. Sci. Transl. Med. 4, 150ra122. 10.1126/scitranslmed.3004291 22956200 PMC3654377

[B57] Schnell A. Chappuis S. Schmutz I. Brai E. Ripperger J. A. Schaad O. (2014). The nuclear receptor REV-ERB*α* regulates *Fabp7* and modulates adult hippocampal neurogenesis. PLoS ONE 9:e99883. 10.1371/journal.pone.0099883 24932636 PMC4059695

[B58] Skene D. J. Swaab D. F. (2003). Melatonin rhythmicity: effect of age and Alzheimer’s disease. Exp. Gerontol. 38, 199–206 10.1016/S0531-5565(02)00198-5 12543278

[B59] Spira A. P. Gamaldo A. A. An Y. Wu M. N. Simonsick E. M. Bilgel M. (2013). Self-reported sleep and *β*-amyloid deposition in community-dwelling older adults. JAMA Neurol. 70, 1537–1543. 10.1001/jamaneurol.2013.4258 24145859 PMC3918480

[B60] Sterniczuk R. Dyck R. H. Laferla F. M. Antle M. C. (2010). Characterization of the 3xTg-AD mouse model of Alzheimer’s disease: part 1. Circadian changes. Brain Res. 1348, 139–148. 10.1016/j.brainres.2010.05.013 20471965

[B61] Swaab D. F. Fliers E. Partiman T. S. (1985). The suprachiasmatic nucleus of the human brain in relation to sex, age and senile dementia. Brain Res. 342, 37–44 10.1016/0006-8993(85)91350-2 4041816

[B62] Tranah G. J. Blackwell T. Stone K. L. Ancoli-Israel S. Paudel M. L. Ensrud K. E. (2011). Circadian activity rhythms and risk of incident dementia and mild cognitive impairment in older women. Ann. Neurol. 70, 722–732. 10.1002/ana.22468 22162057 PMC3244839

[B63] Valnegri P. Khelfaoui M. Dorseuil O. Bassani S. Lagneaux C. Gianfelice A. (2011). A circadian clock in hippocampus is regulated by interaction between oligophrenin-1 and Rev-erb*α*. Nat. Neurosci. 14, 1293–1301. 10.1038/nn.2911 21874017

[B64] Videnovic A. Golombek D. (2013). Circadian and sleep disorders in Parkinson’s disease. Exp. Neurol. 243, 45–56. 10.1016/j.expneurol.2012.08.018 22935723 PMC3666169

[B65] Videnovic A. Lazar A. S. Barker R. A. Overeem S. (2014a). ‘The clocks that time us’-circadian rhythms in neurodegenerative disorders. Nat. Rev. Neurol. 10, 683–693. 10.1038/nrneurol.2014.206 25385339 PMC4344830

[B66] Videnovic A. Noble C. Reid K. J. Peng J. Turek F. W. Marconi A. (2014b). Circadian melatonin rhythm and excessive daytime sleepiness in Parkinson disease. JAMA Neurol. 71, 463–469. 10.1001/jamaneurol.2013.6239 24566763 PMC4078989

[B67] Wisor J. P. Edgar D. M. Yesavage J. Ryan H. S. Mccormick C. M. Lapustea N. (2005). Sleep and circadian abnormalities in a transgenic mouse model of Alzheimer’s disease: a role for cholinergic transmission. Neuroscience 131, 375–385. 10.1016/j.neuroscience.2004.11.018 15708480

[B68] Witting W. Kwa I. H. Eikelenboom P. Mirmiran M. Swaab D. F. (1990). Alterations in the circadian rest-activity rhythm in aging and Alzheimer’s disease. Biol. Psychiatry 27, 563–572 10.1016/0006-3223(90)90523-5 2322616

[B69] Wu Y. H. Fischer D. F. Kalsbeek A. Garidou-Boof M. L. Van Der Vliet J. Van Heijningen C. (2006). Pineal clock gene oscillation is disturbed in Alzheimer’s disease, due to functional disconnection from the “master clock.” FASEB J. 20, 1874–1876. 10.1096/fj.05-4446fje 16818472

[B70] Yamaguchi Y. Suzuki T. Mizoro Y. Kori H. Okada K. Chen Y. (2013). Mice genetically deficient in vasopressin V1a and V1b receptors are resistant to jet lag. Science 342, 85–90. 10.1126/science.1238599 24092737

[B71] Yang Y. K. Peng X. D. Li Y. H. Wang Z. R. Chang-Quan H. Hui W. (2013). The polymorphism of CLOCK gene 3111T/C C>T is associated with susceptibility of Alzheimer disease in Chinese population. J. Investig. Med. 61, 1084–1087. 10.231/JIM.0b013e31829f91c0 23912676

[B72] Zhang R. Lahens N. F. Ballance H. I. Hughes M. E. Hogenesch J. B. (2014). A circadian gene expression atlas in mammals: implications for biology and medicine. Proc. Natl. Acad. Sci. U.S.A. 111, 16219–16224. 10.1073/pnas.1408886111 25349387 PMC4234565

[B73] Zhou J. N. Hofman M. A. Swaab D. F. (1995). VIP neurons in the human SCN in relation to sex, age, and Alzheimer’s disease. Neurobiol. Aging 16, 571–576 10.1016/0197-4580(95)00043-E 8544907

